# Effectiveness of *attexis*, a digital intervention based on cognitive behavioral therapy for adults with ADHD: a randomized controlled trial

**DOI:** 10.1017/S0033291726103390

**Published:** 2026-03-11

**Authors:** Roberto D’Amelio, Linda T. Betz, Sarah M. Jow, Wolfgang Retz, Alexandra Philipsen, Jan Philipp Klein, Eva Fassbinder, Gitta A. Jacob, Petra Retz-Junginger

**Affiliations:** 1 Institute for Forensic Psychology and Psychiatry, Saarland University Medical Center, Homburg, Germany; 2 Gaia AG, Hamburg, Germany; 3 Private Practice for Psychotherapy, Hamburg, Germany; 4 Department for Psychiatry and Psychotherapy, University Medical Center Mainz, Mainz, Germany; 5 Department of Psychiatry and Psychotherapy, University Hospital Bonn, Bonn, Germany; 6 Department of Psychiatry, Psychosomatics, and Psychotherapy, University of Lübeck, Lübeck, Germany; 7 Department of Psychiatry and Psychotherapy, Christian-Albrechts-Universität zu Kiel, Kiel, Germany

**Keywords:** digital therapeutic, digital health, online intervention, pragmatic trial, self-management, self-guided psychotherapy, psychoeducation, mHealth, executive functioning

## Abstract

Access to evidence-based psychosocial interventions for adults with attention-deficit/hyperactivity disorder (ADHD) remains limited, despite strong patient demand for nonpharmacological options such as cognitive behavioral therapy (CBT). Digital interventions may offer a scalable, low-threshold solution to meet this need and complement existing care. This pragmatic randomized controlled trial evaluated the effectiveness of *attexis*, a fully self-guided digital intervention based on CBT and mindfulness principles, as an adjunct to treatment as usual (TAU). A total of 337 adults with confirmed ADHD were randomized to either *attexis* + TAU or TAU alone. The primary outcome was ADHD symptom severity (Adult ADHD Self-Report Scale total score) at 3 months post-randomization (T1). Secondary outcomes included functional impairment, depressive symptoms, self-esteem, and health-related quality of life. Follow-up was conducted at 6 months (T2). Intent-to-treat analyses showed significantly lower ADHD symptom severity in the intervention group at T1 (baseline-adjusted mean difference = −5.0 points; *d* = 0.85, *p* < .001). Significant improvements were also observed across all secondary outcomes, and effects remained stable at T2. Responder analyses confirmed the clinical relevance of the findings. Subgroup analyses demonstrated consistent effects across sex, medication use, psychotherapy status, and treatment changes. No adverse events related to *attexis* were reported. *attexis* was effective in reducing ADHD symptoms and improving a broad range of functional and psychosocial outcomes. As a safe, low-threshold, fully self-guided intervention, it may serve as a valuable adjunct to routine care and help address existing gaps in access to psychosocial treatment for adults with ADHD.

## Introduction

Attention-deficit/hyperactivity disorder (ADHD) affects between 2.5% and 6.8% of adults globally, with an estimated average prevalence of 3.3% in high-income countries (Fayyad et al., 2017; Simon et al., 2009; Song et al., 2021). In Germany, however, estimates based on retrospective claims data from statutory health insurance are substantially lower, at just 0.7%, suggesting marked and longstanding underrecognition of adult ADHD (Libutzki et al., 2019; Philipsen & Döpfner, 2020; Schlander et al., 2007). This is particularly concerning given the high burden of the disorder: Adults with ADHD often face substantial difficulties affecting multiple domains of adult life, including academic and occupational functioning, management of everyday responsibilities such as finances, and interpersonal relationships (de Zwaan et al., 2012; Fuermaier et al., 2021; Koerts et al., 2021). These difficulties are often accompanied by a reduced quality of life extending well into older adulthood (Thorell, Holst, & Sjöwall, 2019), as well as psychological comorbidities, such as depression and anxiety, further exacerbating personal and societal burden (Faraone et al., 2024; Fayyad et al., 2017; Libutzki et al., 2019).

Current European clinical guidelines emphasize a multimodal approach to treating adult ADHD, combining pharmacological and psychosocial interventions to address both core symptoms as well as associated functional and psychosocial impairments (AWMF, 2017; Kooij et al., 2019; NICE, 2018). The German evidence- and consensus-based guideline (AWMF, 2017) recommends psychosocial interventions, particularly in cases of insufficient response to pharmacotherapy, contraindications to medication, poor tolerability, or patient’s decision against pharmacological treatment. Additional indications include adults later in life needing support with diagnosis acceptance and treatment initiation, as well as cases of mild symptomatology where psychosocial treatment is considered sufficient to address low to moderate functional impairments (AWMF, 2017).

Among available psychosocial interventions, psychoeducation and cognitive behavioral therapy (CBT) have demonstrated effectiveness in reducing ADHD symptom severity and associated disorder burden (Hoxhaj et al., 2018; Knouse, Teller, & Brooks, 2017; Liu, Hua, Lu, & Goh, 2023; Nimmo-Smith et al., 2020; Philipsen et al., 2015; Young, Moghaddam, & Tickle, 2020). Accordingly, clinical guidelines endorse both approaches, with psychoeducation recommended as the basis of standard care (AWMF, 2017; Kooij et al., 2019). However, access to qualified care in Germany remains limited: clinicians with expertise in adult ADHD are scarce (Schlander et al., 2007; Schneider et al., 2023), and waiting times for psychotherapy exceed several months (Kruse et al., 2024). As a result, ~40% of adults with ADHD receive no psychotherapy within the first year after diagnosis, and among those who do, 30% receive only probatory sessions (Libutzki et al., 2019).

Digital interventions have demonstrated robust effectiveness across various psychiatric conditions (Andersson, 2016; Karyotaki et al., 2021; Pauley et al., 2023) and are therefore considered a promising strategy to help bridge the treatment gap in adult ADHD. To date, five small-scale randomized controlled trials (RCTs) have evaluated the effectiveness of digital interventions in reducing core ADHD symptom severity, reporting effect sizes between *d* = 0.42 and 1.21 (Kenter, Gjestad, Lundervold, & Nordgreen, 2023; Moëll et al., 2015; Nasri et al., 2023; Pettersson, Söderström, Edlund-Söderström, & Nilsson, 2017; Selaskowski et al., 2022). Despite growing evidence for their effectiveness, digital interventions have not yet been integrated into the routine care of adult ADHD in Germany, and no program is currently approved for reimbursement by statutory health insurance under the *Digital Health Application* scheme.

Against this background, we developed *attexis*, a fully self-guided digital intervention grounded in CBT and mindfulness principles. Thus, *attexis* requires no professional oversight and is accessible online at the user’s convenience. The program provides personalized psychoeducation and therapeutic exercises tailored to individual needs and preferences. Features such as adaptive content delivery, automated reminders, and progress tracking are integrated to support user engagement and motivation.

The goal of the present pragmatic RCT was to evaluate the effectiveness of *attexis* when used as an adjunct to existing treatment pathways. To this end, we compared the addition of *attexis* to treatment as usual (TAU) to a TAU-only control group. TAU reflects the heterogeneous and unstandardized care typically received by adults with ADHD in routine practice in Germany, including pharmacological and nonpharmacological interventions, as well as no formal treatment. The primary endpoint was the severity of ADHD symptoms after 3 months. In addition, we assessed secondary endpoints reflecting functional and psychosocial outcomes relevant to patients, including functional impairment, depressive symptoms, self-esteem, and quality of life. We hypothesized that participants receiving *attexis* in addition to TAU would show greater improvements across these outcomes compared to those receiving TAU alone. Additional follow-up data were collected at 6 months to evaluate the durability of treatment effects.

## Methods

### Procedures

The study “Researching the Effectiveness of attexis, a Digital Health Application for Adults with Attention Deficit Hyperactivity Disorder – a Randomized Controlled Trial (READ-ADHD)” was approved by the Ethics Committee of the Medical Chamber Hamburg (reference number 2023–101052-BO-ff), and all procedures complied with the Declaration of Helsinki and General Data Protection Regulation.

Before recruitment, the study was registered in an international clinical trials registry (ClinicalTrials.gov ID: NCT06221930). German-speaking participants were recruited from March 2024 to June 2024 via an online advertising campaign using targeted Google Ads to reach adults with ADHD symptoms. Interested individuals were directed to a dedicated study website containing detailed study information and an option to register their interest.

After providing electronic informed consent, participants completed an initial online screening survey. Those who screened positively were invited to a structured diagnostic interview conducted by telephone. Trained psychological staff administered the German version of the DIVA-5 (Kooij, Francken, Bron, & Wynchank, 2019) to confirm an ADHD diagnosis and the Mini-DIPS (Margraf, Cwik, Pflug, & Schneider, 2017) to assess relevant current psychiatric comorbidities. Ambiguous cases were reviewed in regular supervision meetings with GAJ. Final eligibility was determined by the study physician, SMJ, following a comprehensive review of inclusion and exclusion criteria.

Eligible participants were randomized in a 1:1 ratio to either the intervention group (*attexis* + TAU) or the control group (TAU only), using an automated and concealed allocation system using computer-generated random numbers (simulating a digital coin toss) to perform simple randomization. Allocation was triggered automatically by the study platform upon completion of eligibility verification. Due to the nature of the pragmatic design, participants were not blinded to group allocation. As all outcomes were collected via self-report, no outcome assessors were involved, and blinding was therefore not applicable. Participants in the intervention group received immediate access to *attexis*, while control group participants were offered access upon study completion. The study was designed as a pragmatic trial, aiming to approximate real-world conditions without restricting concurrent treatment options: TAU permitted participants in both groups to continue, modify, or discontinue any concurrent treatments, including psychotherapy and pharmacotherapy. Planned treatment changes in the upcoming 3 months were an exclusion criterion at baseline, in order to better attribute observed effects to *attexis.* Unplanned treatment changes that occurred during the course of the study were allowed and documented, reflecting the pragmatic nature of the trial.

Participants completed outcome assessments online at 3 months (T1, primary time point for evaluating effectiveness) and 6 months (T2, follow-up to assess the durability of effects over time). All study procedures were conducted fully remotely; questionnaires were administered online, and diagnostic interviews were conducted by telephone. Participants received a €10 gift voucher for every completed follow-up assessment.

The required sample size was determined a priori based on the primary outcome; see Supplementary Method 1 for details.

### Inclusion and exclusion criteria

Participants of all sexes and genders were eligible for inclusion if they were between 18 and 65 years of age and had a diagnosis of ADHD confirmed via the structured diagnostic interview DIVA-5. To meet the symptom severity threshold, participants were required to score ≥ 17 on either the inattention or the hyperactivity/impulsivity subscale of the Adult ADHD Self-Report Scale v1.1 (ASRS), in line with thresholds used in prior research on digital interventions for ADHD (Kenter, Gjestad, Lundervold, & Nordgreen, 2023; Moëll et al., 2015). Additional inclusion criteria were stable treatment status (including psychotherapy, medication, or no treatment) for at least 30 days before enrollment, sufficient knowledge of the German language, and provision of informed consent.

Participants were excluded if they had a lifetime diagnosis of a severe psychiatric disorder, including severe affective disorder, psychotic disorder, autism spectrum disorder, borderline or antisocial personality disorder, substance use disorder, or suicidality. Individuals were also excluded if they planned to change their current treatment regimen (e.g., psychotherapy or medication) within the 3 months following enrollment.

### Intervention

*attexis* is a self-guided online intervention based on cognitive-behavioral and mindfulness principles, developed to support adults with ADHD. *attexis* combines psychoeducation, therapeutic exercises, and personalized techniques tailored to the user’s reported needs and preferences (see Table 1). A central feature is its use of “simulated dialogues,” in which users interact with brief text passages by selecting response options that guide the flow of information and tailor the experience (see Supplementary Figure for example screenshots). In this context, *attexis* specifically addresses the interpersonal needs of patients with ADHD. For instance, depending on the individual’s attentional state, it may offer to repeat certain topics or suggest humorous or unexpected exercises to help sustain attention. This format supports personalization and sustained engagement. Thus, the interaction itself is highly flexible and individualized. However, the main topics covered by the program are set in a fixed order. The program begins with psychoeducation, for example, about the diagnosis and the concept of neurodiversity, followed by techniques to improve attention. Only then are topics such as self-image addressed. This is consistent with the approach used in manualized psychotherapies. The program also includes simple homework tasks, flexible pacing, and automated reminders to encourage continued use. In addition to its core dialogues, *attexis* provides Supplementary Materials such as guided audio exercises, PDF worksheets, and summary sheets. Users may also opt to receive motivational messages via SMS or email, and self-monitoring tools are included to support behavioral tracking and progress monitoring over time.

**Table 1.** Content of *attexis*

Topic	Content focus
Psychoeducation	Comprehensive information on ADHD diagnosis, symptomatology, and associated challenges; introduction to the neurodiversity perspective to foster self-understanding and reduce internalized stigma.
Mindfulness	Use of mindfulness to more quickly identify and interrupt problems related to attention and impulsivity; in this context, mindfulness is also practiced repeatedly as part of the program.
Problem identification	Guided exploration of individual difficulties and life areas affected by ADHD to personalize the therapeutic approach and increase relevance.
Stigma and gender-specific aspects	Discussion of public and internalized stigma, as well as gender-specific manifestations of ADHD and their implications for self-image and care-seeking behavior.
Attention	Analysis of individual “attention disruptors” and “attention sustainers”; techniques to enhance focus and compensate for inattention in daily life.
Impulsivity and emotion regulation	Development of strategies to interrupt impulsive actions; promotion of physical activity for emotional regulation and behavioral stabilization.
Problem solving	Training in structured approaches to problem-solving using techniques such as SMART goal setting and the Eisenhower matrix.
Self-esteem and self-image	Processing of negative childhood experiences related to ADHD; resource-oriented reframing of ADHD traits; use of imagery-based techniques to strengthen a positive self-concept.
Resource orientation	Use of positive psychology principles and exercises to identify personal strengths and apply them effectively in everyday life situations.

### Measures

Baseline data, as well as follow-up data at 3 months (T1) and 6 months (T2) post-randomization, were collected via a secure and encrypted online survey platform (*LimeSurvey*). All measures were based on self-report. Participants were invited to complete each assessment via email, and nonresponders received up to three reminders. For follow-up assessments, additional contact methods for reminders, including SMS and telephone calls, were employed to maximize response rates.

#### Primary endpoint

The primary endpoint was the ASRS total score, a widely used 18-item patient-reported outcome measure assessing core ADHD symptoms (Kessler et al., 2005), evaluated at T1 (3 months post-randomization). The validated German version of the ASRS has demonstrated good psychometric properties in adults with ADHD (Mörstedt, Corbisiero, & Stieglitz, 2016).

#### Secondary endpoints

Secondary outcomes included: (1) functional impairment, assessed using the Work and Social Adjustment Scale (Heissel et al., 2021; Lundqvist et al., 2024; Mundt, Marks, Shear, & Greist, 2002); (2) depressive symptoms, measured by the Patient Health Questionnaire-9 (Hennig et al., 2017; Kliem et al., 2024; Martin, Rief, Klaiberg, & Braehler, 2006); (3) self-esteem, assessed with the Rosenberg Self-Esteem Scale (Masuch et al., 2019; Rosenberg, 1965; Roth, Decker, Herzberg, & Brähler, 2008; von Collani & Herzberg, 2003); and (4) quality of life, measured by the Assessment of Quality of Life - 8 Dimensions (Richardson, Iezzi, Khan, & Maxwell, 2014; Richardson, Khan, Iezzi, & Maxwell, 2012).

#### Comorbidities and user satisfaction

Relevant sections from the Mini-DIPS were used to assess current psychiatric comorbidities in the sample (Margraf, Cwik, Pflug, & Schneider, 2017). To assess satisfaction with *attexis*, participants were asked to rate how likely they were to recommend *attexis* to a friend or colleague on a numeric rating scale from 0 (not likely at all) to 10 (extremely likely). Moreover, subjective improvement of ADHD symptoms as well as subjective improvement in the impact of ADHD on daily activities in the last 3 months (from T0 to T1 and from T1 to T2) was evaluated with the Patient Global Impression of Change scale (PGIC; Guy, 1976).

#### Adverse events

Given that *attexis* is a Conformité Européenne (CE)-marked Class I medical device, it is inherently associated with a very low risk profile. Nonetheless, adverse events were monitored throughout the study and operationalized as any unplanned or emergency outpatient or inpatient treatment reported for the preceding 3 months. Participants were asked to report such events at each follow-up assessment (T1 and T2). In addition, any events potentially related to the use of the intervention were documented as adverse device effects. All reported events were reviewed for potential causal association with *attexis.*

### Statistical analyses

All analyses followed a prespecified analysis plan and were conducted using *R*, version 4.4.1 (R Core Team, 2024). The primary analyses applied the intent-to-treat (ITT) principle under a missing at random assumption, using analysis of covariance adjusted for baseline values. Results are reported as baseline-adjusted mean differences with 95% confidence intervals (CIs) and standardized effect sizes (Cohen’s *d* based on estimated marginal means). Missing data were handled via bootstrapped multiple imputation (von Hippel & Bartlett, 2021). As a sensitivity analysis, we applied jump-to-reference (J2R) imputation, a conservative method assuming intervention dropouts follow the control trajectory (Bartlett, 2023). Per-protocol (PP) analyses, defined as including only intervention participants who had registered to use *attexis*, alongside all participants in the control group, applied the same statistical procedures as the ITT analyses. All analyses were repeated at T2 to assess the durability of effects. Clinical relevance was evaluated using responder analyses. For the primary endpoint, responders were defined as participants who showed a ≥30% reduction in the ASRS total score from baseline to T1 (Buitelaar, Montgomery, & van Zwieten-Boot, 2003). For secondary endpoints, responder criteria were based on predefined minimal clinically important differences (MCIDs), or on the reliable change index (RCI) where no MCID was available. Group differences in responder rates were analyzed using *χ*^2^ tests and odds ratios (ORs). As a safety analysis, we assessed symptom worsening, defined as any increase in the ASRS total score from baseline to T1. Subgroup analyses assessed potential treatment moderators (sex, psychotherapy, psychotropic medication, and treatment changes). Statistical tests were considered significant at *p* < .05 (two-sided). A gatekeeping testing strategy was applied to control for multiplicity. Full details of the statistical analysis are provided in Supplementary Method 2.

## Results

### Recruitment and retention overview

**Figure 1.**
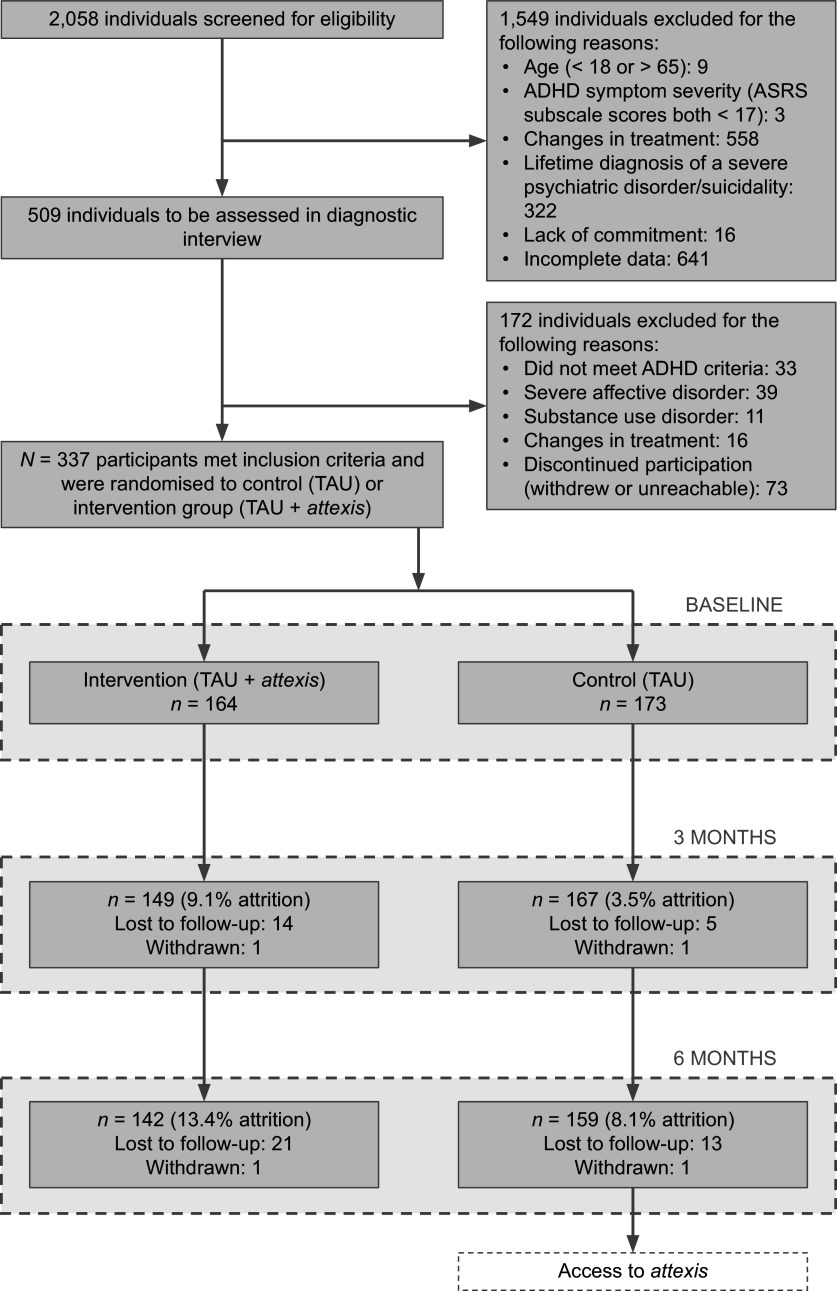
Flow of participants through the study. *Note*: ASRS, ‘Adult ADHD Self-Report Scale’; TAU, ‘treatment as usual’.

A total of 2,058 individuals were screened for eligibility, of whom 337 met all inclusion criteria and were randomized to the intervention group (*n* = 164) or the control group (*n* = 173) (see Figure 1). The slight imbalance in group sizes is consistent with expected variation under simple randomization. Attrition rates at 3 months were 9.1% in the intervention group and 3.5% in the control group; at 6 months, attrition increased slightly to 13.4% and 8.1%, respectively. Following the predefined criteria for inclusion in the PP analyses, 163 out of 164 participants (99.4%) in the intervention group had activated the voucher to use *attexis.* The resulting PP dataset comprised 336 participants: 163 in the intervention group and all 173 in the control group.

### Baseline characteristics

Table 2 presents baseline characteristics of the study sample. The mean age was ~38 years, with 71% reporting female sex. Most participants were in a relationship and had a higher level of education; over half held a university degree. Employment was common, with 42% working full-time and 33% part-time. The majority identified as White (93%), with smaller proportions reporting Middle Eastern (4.5%), Latin American (2.1%), or Black (1.2%) ethnicity. Current comorbid psychiatric conditions were frequent: 27% met diagnostic criteria for major depressive disorder, and 12% for social anxiety disorder. About 24% were currently in psychotherapy, and 36% were taking psychotropic medication, most commonly psychostimulants (28%).

**Table 2.** Subject demographics and clinical characteristics at baseline. Values represent mean (SD) unless stated otherwise

	Control	*attexis*	Total
	*n* = 173	*n* = 164	*n* = 337
Age	37.47 (9.88)	37.82 (9.31)	37.64 (9.59)
Sex (*n* [%])			
Female	118 (68.2)	121 (73.8)	239 (70.9)
Male	55 (31.8)	43 (26.2)	98 (29.1)
Intersexual	0 (0)	0 (0)	0 (0)
Relationship status (*n* [%])			
Single	40 (23.1)	43 (26.2)	83 (24.6)
In a relationship	133 (76.9)	121 (73.8)	254 (75.4)
Family situation (*n* [%])			
Never married	93 (53.8)	84 (51.2)	177 (52.5)
Married/registered civil partnership	72 (41.6)	70 (42.7)	142 (42.1)
Divorced/registered partnership annulled	8 (4.6)	10 (6.1)	18 (5.3)
Widowed/registered partner deceased	0 (0)	0 (0)	0 (0)
Education (*n* [%])			
Lower secondary school certificate	3 (1.7)	1 (0.6)	4 (1.2)
Intermediate secondary school certificate	12 (6.9)	9 (5.5)	21 (6.2)
University of Applied Sciences entrance qualification	20 (11.6)	12 (7.3)	32 (9.5)
General university entrance qualification	22 (12.7)	13 (7.9)	35 (10.4)
Completed vocational training	29 (16.8)	32 (19.5)	61 (18.1)
Completed university studies	87 (50.3)	97 (59.1)	184 (54.6)
Employment (*n* [%])			
Not employed	27 (15.6)	15 (9.1)	42 (12.5)
Employed irregularly	3 (1.7)	3 (1.8)	6 (1.8)
Marginal employment	5 (2.9)	7 (4.3)	12 (3.6)
Employed part-time	53 (30.6)	58 (35.4)	111 (32.9)
Employed full-time	71 (41.0)	71 (43.3)	142 (42.1)
In vocational training	5 (2.9)	1 (0.6)	6 (1.8)
On parental leave	6 (3.5)	6 (3.7)	12 (3.6)
In re-training	3 (1.7)	3 (1.8)	6 (1.8)
Ethnicity (multiple answers possible; *n* [%])			
White	162 (93.6)	151 (92.1)	313 (92.9)
Black	1 (0.6)	3 (1.8)	4 (1.2)
Middle Eastern	7 (4.0)	8 (4.9)	15 (4.5)
Latin American	3 (1.7)	4 (2.4)	7 (2.1)
South East Asian	2 (1.2)	1 (0.6)	3 (0.9)
East Asian	0	0	0
South Asian	0	0	0
Unknown	1 (0.6)	0 (0.0)	1 (0.3)
Prefer not to say	2 (1.2)	1 (0.6)	3 (0.9)
Sick days (last 3 months; *n* [%])			
0 days	81 (46.8)	79 (48.2)	160 (47.5)
1–5 days	43 (24.9)	45 (27.4)	88 (26.1)
6–10 days	17 (9.8)	16 (9.8)	33 (9.8)
10+ days	32 (18.5)	24 (14.6)	56 (16.6)
Sick pay days (last 3 months; *n* [%])			
0 days	158 (91.3)	152 (92.7)	310 (92.0)
1–5 days	6 (3.5)	5 (3.0)	11 (3.3)
6–10 days	1 (0.6)	2 (1.2)	3 (0.9)
10+ days	8 (4.6)	5 (3.0)	13 (3.9)
Prior ADHD diagnosis (*n* [%])	74 (42.8)	76 (46.3)	150 (44.5)
Age at diagnosis (in individuals with prior ADHD diagnosis; in years)	32.86 (13.65)	33.51 (11.82)	33.19 (12.72)
Current psychiatric diagnoses (Mini-DIPS) (multiple answers possible; *n* [%])			
Panic disorder	3 (1.7)	4 (2.4)	7 (2.1)
Agoraphobia	4 (2.3)	7 (4.3)	11 (3.3)
Specific phobia	16 (9.2)	22 (13.4)	38 (11.3)
Social anxiety disorder	21 (12.1)	18 (11.0)	39 (11.6)
Generalized anxiety disorder	6 (3.5)	8 (4.9)	14 (4.2)
Bipolar disorder	0 (0.0)	1 (0.6)	1 (0.3)
Major depressive disorder	43 (24.9)	47 (28.7)	90 (26.7)
Persistent depressive disorder	3 (1.7)	1 (0.6)	4 (1.2)
Hypersomnia	5 (2.9)	3 (1.8)	8 (2.4)
Insomnia	15 (8.7)	9 (5.5)	24 (7.1)
Currently in psychotherapy (*n* [%])	43 (24.9)	36 (22.0)	79 (23.4)
Number of psychotherapy sessions^a^	1.47 (3.10)	1.39 (3.12)	1.43 (3.11)
Currently treated by an ADHD specialist	5 (2.9)	7 (4.3)	12 (3.6)
Ever in psychotherapy (*n* [%])	95 (54.9)	103 (62.8)	198 (58.8)
Self-medicating (*n* [%])	13 (7.5)	13 (7.9)	26 (7.7)
Currently taking any psychotropic medication^b^ (*n* [%])	59 (34.1)	63 (38.4)	122 (36.2)
Regular medication (multiple answers possible; *n* [%])			
Antipsychotics	1 (0.6)	0 (0.0)	1 (0.3)
Anxiolytics	0 (0.0)	0 (0.0)	0 (0.0)
Hypnotics and sedatives	3 (1.7)	0 (0.0)	3 (0.9)
Antidepressants	15 (8.7)	20 (12.2)	35 (10.4)
Psychostimulants	41 (23.7)	52 (31.7)	93 (27.6)
Medication as needed (multiple answers possible; *n* [%])			
Antipsychotics	0 (0.0)	0 (0.0)	0 (0.0)
Anxiolytics	0 (0.0)	0 (0.0)	0 (0.0)
Hypnotics and sedatives	4 (2.3)	0 (0.0)	4 (1.2)
Antidepressants	1 (0.6)	2 (1.2)	3 (0.9)
Psychostimulants	14 (8.1)	5 (3.0)	19 (5.6)
ASRS total score	52.7 (7.1)	52.2 (7.4)	52.5 (7.3)
WSAS total score	22.9 (7.1)	23.3 (7.3)	23.1 (7.2)
PHQ-9 total score	11.8 (4.4)	11.7 (5.0)	11.8 (4.7)
RSES total score	17.2 (5.7)	17.1 (5.9)	17.2 (5.8)
AQoL-8D total score	63.3 (9.5)	63.1 (10.1)	63.2 (9.8)

*Note:* AQoL-8D, Assessment of Quality of Life - 8 Dimensions; ASRS, Adult ADHD Self-Report Scale; PHQ-9, Patient Health Questionnaire-9; RSES, Rosenberg Self-Esteem Scale; WSAS, Work and Social Adjustment Scale.

a Calculated across all participants, including those with zero sessions.

b Including medications classified under the Anatomical Therapeutic Chemical (ATC) system as N05 (psycholeptics) and N06 (psychoanaleptics).

### Missingness

Group differences in baseline characteristics between participants who completed the primary endpoint assessment at T1 (*N* = 316) and those who dropped out for any reason (*N* = 21) are reported in Supplementary Table 1. Compared to completers, dropouts were significantly younger, less likely to identify as White, more likely to be treated by an ADHD specialist, and more likely to be taking psychotropic medication. They also reported higher baseline levels of ADHD symptoms and functional impairment.

### Treatment effects

#### Primary endpoint: ADHD symptom severity

**Figure 2.**
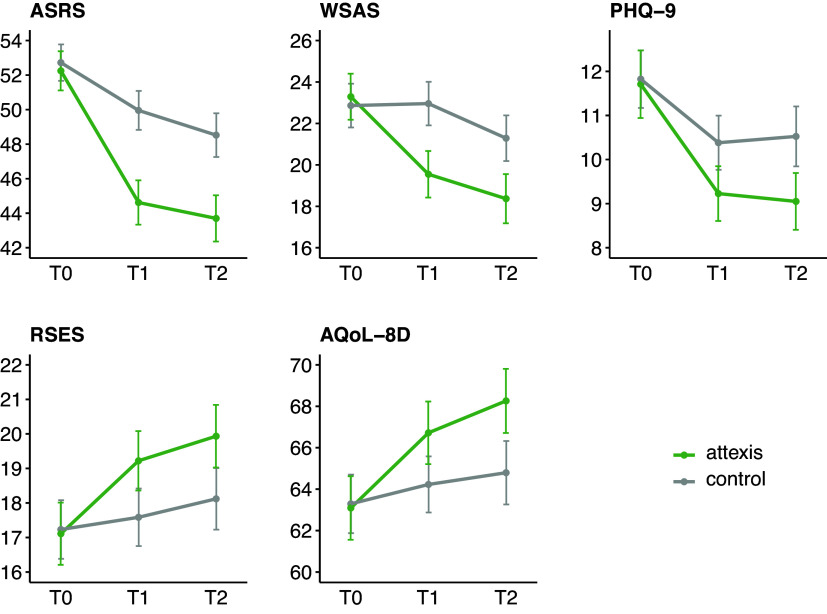
Symptom course on primary and secondary outcomes (means ±95% confidence interval) using intent-to-treat analyses with multiple imputation for missing data. Plotted values represent total scores. *Note:* ASRS, ‘Adult ADHD Self-Report Scale’; AQoL-8D, ‘Assessment of Quality of Life - 8 Dimensions’; PHQ-9, ‘Patient Health Questionnaire-9’; RSES, ‘Rosenberg Self-Esteem Scale’; WSAS, ‘Work and Social Adjustment Scale’.

After 3 months, participants in the *attexis* + TAU group showed significantly lower ADHD symptom severity compared to the TAU-only group (see Table 3 and Figure 2). The baseline-adjusted mean difference on the ASRS total score was −5.0 points (95% CI = [−6.4, −3.6], *p* < .001; *d* = 0.85). Comparable effects were found in the J2R sensitivity analysis (−4.4 points, 95% CI [−5.7, −3.2], *p* < .001; *d* = 0.75) and the PP analysis (−5.0 points, 95% CI = [−6.4, −3.6], *p* < .001; *d* = 0.86). After 6 months, the ITT analysis confirmed a continued intervention effect (−4.5 points, 95% CI = [−6.2, −2.9], *p* < .001; *d* = 0.61). The J2R and PP analyses yielded comparable results (see Supplementary Tables 2 and 3), confirming the robustness of the treatment effect in relation to different assumptions concerning missing outcome data.

**Table 3.** Results of primary and secondary endpoints for ITT analyses

	Time	Control			*attexis*			ANCOVA		
		*n*	Mean	SD	*n*	Mean	SD	Treatment effect^a^ (95% CI)	*p*-value	Cohen’s *d* (95% CI)^b^
ASRS	T0	173	52.7	7.1	164	52.2	7.4	–	–	–
	T1	173	50.0	7.6	164	44.6	8.4	−5.0 (−6.4, −3.6)	< .001	0.85 (0.62, 1.08)
	T2	173	48.5	8.5	164	43.7	8.7	−4.5 (−6.2, −2.9)	< .001	0.61 (0.39, 0.83)
WSAS	T0	173	22.9	7.1	164	23.3	7.2	–	–	–
	T1	173	23.0	7.0	164	19.5	7.3	−3.7 (−5.0, −2.3)	< .001	0.61 (0.38, 0.84)
	T2	173	21.3	7.4	164	18.4	7.8	−3.1 (−4.6, −1.6)	< .001	0.47 (0.24, 0.7)
PHQ-9	T0	173	11.8	4.4	164	11.7	5.0	–	–	–
	T1	173	10.4	4.1	164	9.2	4.1	−1.1 (−1.8, −0.4)	.003	0.32 (0.10, 0.54)
	T2	173	10.5	4.6	164	9.1	4.2	−1.4 (−2.3, −0.5)	.002	0.36 (0.14, 0.57)
RSES	T0	173	17.2	5.7	164	17.1	5.9	–	–	–
	T1	173	17.6	5.6	164	19.2	5.6	1.7 (0.9, 2.5)	< .001	0.48 (0.25, 0.70)
	T2	173	18.1	6.0	164	19.9	5.9	1.9 (0.9, 2.9)	< .001	0.43 (0.21, 0.65)
AQoL-8D	T0	173	63.3	9.5	164	63.1	10.0	–	–	–
	T1	173	64.2	9.1	164	66.7	9.9	2.6 (1.3, 4.0)	< .001	0.44 (0.22, 0.66)
	T2	173	64.8	10.3	164	68.3	10.1	3.6 (2.0, 5.3)	< .001	0.47 (0.26, 0.69)

*Note:* AQoL-8D, Assessment of Quality of Life - 8 Dimensions; ASRS, Adult ADHD Self-Report Scale; PHQ-9, Patient Health Questionnaire-9; RSES, Rosenberg Self-Esteem Scale; WSAS, Work and Social Adjustment Scale.

a Between-group difference on the original scale at 3 months (T1) and 6 months (T2), adjusted for baseline scores.

b Based on baseline-adjusted means; positive values show effects in favor of the intervention group.

After 3 months, 11.6% of participants in the intervention group (19/164) met the predefined responder criterion (≥30% reduction in the ASRS total score), compared to 1.2% in the control group (2/173). The difference between groups was statistically significant (*χ*^2^ = 15.67, *p* < .001), corresponding to an OR of 11.2 (95% CI = [2.6, 48.9]). The safety analysis further indicated that symptom worsening (i.e. any increase in ASRS total score from baseline to T1) was observed in 10.7% of participants in the intervention group (16/149) and 24% in the control group (40/167). The difference was statistically significant (*χ*^2^ = 9.43, *p* = .002; OR = 2.6, 95% CI = [1.4, 5.0]), indicating that the use of *attexis* was associated with a lower likelihood of symptom worsening compared to TAU alone.

#### Secondary endpoints

After 3 months, ITT analyses showed significant improvements in the intervention group across all secondary outcomes (see Table 3 and Figure 2), including work and social functioning, depression, self-esteem, and quality of life. Effect sizes ranged from *d* = 0.32 to 0.61. J2R and PP analyses yielded consistent results (see Supplementary Tables 2 and 3).

Responder analyses confirmed the clinical relevance of these effects: significantly more participants in the intervention group showed meaningful improvements in work and social functioning (based on an MCID of 8 points [Zahra et al., 2014]; 26.2% vs. 11.6%; *χ*^2^ = 11.9, *p* < .001; OR = 2.7, 95% CI = [1.5, 4.9]), depression levels (based on an MCID of 5 points [Löwe et al., 2004]; 31.1% vs. 19.7%; *χ*^2^ = 5.85, *p* = .016; OR = 1.8, 95% CI = [1.1, 3.0]), self-esteem (based on the RCI; 20.1% vs. 9.8%; *χ*^2^ = 7.06, *p* = .008; OR = 2.3, 95% CI = [1.2, 4.3]), and quality of life (based on the RCI; 21.3% vs. 9.8%; *χ*^2^ = 8.55, *p* = .003; OR = 2.5, 95% CI = [1.3, 4.6]).

At 6 months, intervention effects continued to be observable across all secondary outcomes (see Table 3 and Figure 2). Effect sizes ranged from *d* = 0.36 to 0.47. These results were consistent in both PP and J2R analyses (see Supplementary Tables 2 and 3).

#### Subgroup analyses

Subgroup analyses showed consistent effectiveness of *attexis* across sex, psychotherapy status at baseline, psychotropic medication use, and treatment changes between T0 and T1 (see Supplementary Tables 4–7).

#### User satisfaction

Mean ratings for the likelihood to recommend the program to a friend or colleague were 6.1 (standard deviation [SD] = 2.9) at T1 and 5.9 (SD = 3.3) at T2, indicating a generally positive evaluation of *attexis.*

Subjective ADHD symptom improvement (assessed via the PGIC) was significantly greater in the intervention group at both T1 and T2 (T1: *d* = 0.56; T2: *d* = 0.55; both *ps* < .001). Similar effects were observed for perceived improvements in daily functioning (T1: *d* = 0.66; T2: *d* = 0.63; both *ps* < .001). In binary ratings, 36.5% of participants in the intervention group reported meaningful perceived functional improvement at T1 versus 9.6% in the control group (*χ*^2^ = 32.9, *p* < .001); at T2, this was 40.1% versus 17.6% (*χ*^2^ = 18.8, *p* < .001).

#### Adverse events

After 3 months, unplanned or emergency medical treatments were reported by 7.8% of participants in the control group (13/167) and 5.4% in the intervention group (8/147), with no significant group difference (*χ*^2^ = 0.69, *p* = .407). A similar pattern was observed after 6 months (control: 6.3%, 10/159; intervention: 4.2%, 6/142; *χ*^2^ = 0.63, *p* = .426). No adverse events were linked to the use of *attexis*, and no adverse device effects were reported.

#### Usage

Registered patients spent an average of 10.9 h (SD = 6.5) in the program up to T1 and 13.3 h (SD = 6.9) up to T2, across an average of 10 (SD = 5.1) and 11 different use days (SD = 6.2), respectively. Changes in ADHD symptom severity from baseline to T1 were not significantly correlated with total hours spent in the program up to T1 (*r* = 0.08, *p* = .292), and showed only a negligible correlation with the number of active days up to T1 (*r* = 0.17, *p* = .044).

## Discussion

This pragmatic RCT evaluated the effectiveness of *attexis*, a fully self-guided digital intervention based on cognitive-behavioral and mindfulness principles for adults with ADHD. Results show that *attexis*, when added to TAU, significantly reduced ADHD symptom severity and improved a range of patient-relevant functional and psychosocial secondary outcomes compared to TAU-only. Effects were robust under conservative assumptions regarding missing outcome data and sustained at the 6-month follow-up, highlighting the long-term potential of the intervention. Responder analyses confirmed the clinical relevance of the intervention effects across all outcomes. Participants reported good satisfaction with *attexis*, along with perceived improvements in ADHD symptoms and their impact on daily life. Importantly, no adverse events were attributed to the use of *attexis.*

In terms of the primary outcome, i.e. ADHD symptom severity after 3 months, *attexis* demonstrated a large effect size (*d* = 0.85). This finding aligns with previous research on digital interventions for adult ADHD, which reported effect sizes ranging from *d* = 0.42 to 1.21 for this outcome (Kenter, Gjestad, Lundervold, & Nordgreen, 2023; Moëll et al., 2015; Nasri et al., 2023; Pettersson, Söderström, Edlund-Söderström, & Nilsson, 2017; Selaskowski et al., 2022). However, all previous studies featured small sample sizes, a common limitation in research on nonpharmacological treatments for adult ADHD (Nimmo-Smith et al., 2020). In contrast, the present study was adequately powered and, to our knowledge, represents the largest RCT to date evaluating a digital intervention for adults with ADHD. Therefore, our study strengthens the evidence base for digital interventions in this population. Notably, the magnitude of the intervention effect falls within the range reported in meta-analyses of face-to-face CBT for adult ADHD (*d* = 0.71–0.98) (Liu, Hua, Lu, & Goh, 2023; Lopez et al., 2018; Young, Moghaddam, & Tickle, 2020). Thus, our results mirror previous meta-analytic findings suggesting that therapeutic content may matter more than delivery format in the therapy of adult ADHD (Knouse, Teller, & Brooks, 2017).

Regarding secondary outcomes, use of *attexis* was linked to consistent benefits in a wide range of functional and psychosocial domains, specifically, occupational and social functioning, depressive symptoms, self-esteem, and quality of life. These outcomes are highly relevant from a patient perspective, as they reflect the real-world burden of ADHD and are closely linked to long-term functioning and well-being (Coghill et al., 2017; Cook, Knight, Hume, & Qureshi, 2014; D’Amelio, Retz, Philipsen, & Rösler, 2009). Despite their clinical relevance and inclusion in guideline recommendations, they remain insufficiently addressed in both routine care and research (AWMF, 2017; Kooij et al., 2019; López-Pinar et al., 2020). This may, in part, be due to the fact that pharmacotherapy, currently the predominant treatment approach for adult ADHD, has shown only limited and inconsistent effects on these broader psychosocial outcome domains (Bellato et al., 2024; Castells, Blanco-Silvente, & Cunill, 2018; Coghill et al., 2017; Lenzi, Cortese, Harris, & Masi, 2018).

Over the 6-month follow-up, we observed relevant dynamics in the evaluated outcomes: While the intervention effects of *attexis* on core ADHD symptoms and functional impairment were maintained at 6 months, improvements in depressive symptoms and quality of life increased further over time. This pattern suggests that as core symptoms decrease, individuals begin to engage more with their environment and implement adaptive coping strategies in daily life. According to CBT models of adult ADHD, such behavioral engagement and cognitive restructuring can, over time, reduce comorbid internalizing symptoms and the broader real-world burden associated with ADHD (López-Pinar et al., 2020; Safren, Sprich, Chulvick, & Otto, 2004).

Overall, our findings address a relevant gap in the current treatment for adults with ADHD. Psychosocial interventions, particularly CBT, are recommended in the treatment of adult ADHD (AWMF, 2017; Kooij et al., 2019), but access remains limited, with long waiting times and a lack of trained professionals representing well-documented barriers in Germany (Kruse et al., 2024; Libutzki et al., 2019; Schneider et al., 2023). This is reflected in our sample, where only 3.6% of participants received care by an ADHD specialist at baseline. Importantly, many adults with ADHD report that pharmacological treatment alone is inadequate and express a desire for psychoeducation and psychosocial support (AWMF, 2017; Matheson et al., 2013). Reflecting this, meta-analytic evidence indicates that combining pharmacotherapy with CBT may lead to improved treatment outcomes in adult ADHD compared to pharmacotherapy alone (Lopez et al., 2018). Easily accessible digital interventions, such as *attexis*, may represent a valuable addition to the treatment of adult ADHD across different clinical contexts, including, as evidenced by subgroup analyses, both as a stand-alone tool and in combination with pharmacotherapy.

## Limitations

Several limitations need to be considered when interpreting our results. First, while ADHD diagnoses were confirmed before inclusion by trained clinicians using structured interviews, all outcome measures were based on self-report, which may be subject to recall or social desirability bias. In line with patient-centered care, however, self-report captures patients’ subjective experience, and the use of validated instruments and objective responder criteria supports the reliability of the findings. Moreover, existing evidence does not suggest that self-reports systematically overestimate treatment effects; for example, a recent meta-analysis comparing self- and clinician-rated outcomes of psychotherapies for depression found that both masked and unmasked clinician ratings yielded larger effects than self-ratings (Miguel et al., 2025). Second, participants were not blinded to group allocation, which, combined with longer treatment time delivered through *attexis* compared to TAU, may have exaggerated intervention effects, for example, via expectancy effects. That said, blinding is generally difficult in psychotherapy research, as the development of credible but ineffective sham interventions poses significant conceptual challenges (Kirsch, Wampold, & Kelley, 2016). Moreover, TAU is considered an appropriate comparator when the primary objective is to establish an intervention’s effectiveness in the context of existing clinical practice, which was also the case in our trial (Freedland et al., 2019). Third, satisfaction ratings suggest that experiences with the program varied. Future qualitative research should examine more in-depth which components were perceived as helpful or lacking by patients. Fourth, attrition was overall low compared to the literature (Linardon & Fuller-Tyszkiewicz, 2020), but slightly higher in the intervention group. While this pattern is common (Assmann et al., 2025; Betz et al., 2024; Specht et al., 2024) and was addressed analytically, underlying reasons for dropout warrant further investigation. Fifth, in absolute terms, the proportion of patients in the intervention group who reached the predefined responder criterion for the primary endpoint was relatively low, albeit significantly higher than in the control group. The corresponding effect size was considerable (OR = 11.2), supporting the clinical relevance of the finding. Finally, generalizability may be limited to digitally savvy individuals willing to engage with a digital solution for the treatment of ADHD symptoms. However, this population likely mirrors those who are most likely to use and benefit from prescribed digital interventions in real-world care settings (Betz et al., 2024).

## Conclusion

In conclusion, this study adds important evidence to the growing field of digital interventions for adult ADHD. *attexis*, a fully self-guided digital intervention based on BT and mindfulness principles, was associated with significant and clinically relevant reductions in ADHD symptom severity and improvements in a broad range of patient-relevant secondary outcomes, including functional impairment, depressive symptoms, self-esteem, and quality of life. The intervention showed consistent effects across diverse subgroups and was well accepted by users. While further research is needed to replicate and extend these findings, particularly in routine care settings, digital interventions like *attexis* may offer a promising treatment option for adults with ADHD, especially in light of limited access to evidence-based psychosocial interventions.

## Supporting information

10.1017/S0033291726103390.sm001 D’Amelio et al. supplementary material D’Amelio et al. supplementary material

## Supplementary material

The supplementary material for this article can be found at http://doi.org/10.1017/S0033291726103390.
