# Supplementary material for: Effectiveness of attexis, a digital intervention based on cognitive behavioral therapy for adults with ADHD: a randomized controlled trial
Source: Psychol Med. 2026 Mar 11;56:e54. doi: 10.1017/S0033291726103390 (PMC13079232; doi:10.1017/S0033291726103390)
Supplement: D’Amelio et al. supplementary material [file S0033291726103390sup001.pdf]

Supplementary Material for

"Effectiveness of *attexis*, a Digital Intervention Based on Cognitive Behavioral Therapy for Adults with ADHD: A Randomized Controlled Trial"

Roberto D'Amelio, Linda T. Betz, Sarah M. Jow, Wolfgang Retz, Alexandra Philipsen, Jan Philipp Klein, Eva Fassbinder, Gitta A. Jacob, Petra Retz-Junginger

Supplementary Figure. Selected screenshots of *attexis* with English translations for illustrative purposes. The software was used exclusively in German during the study; an English version is not currently available.

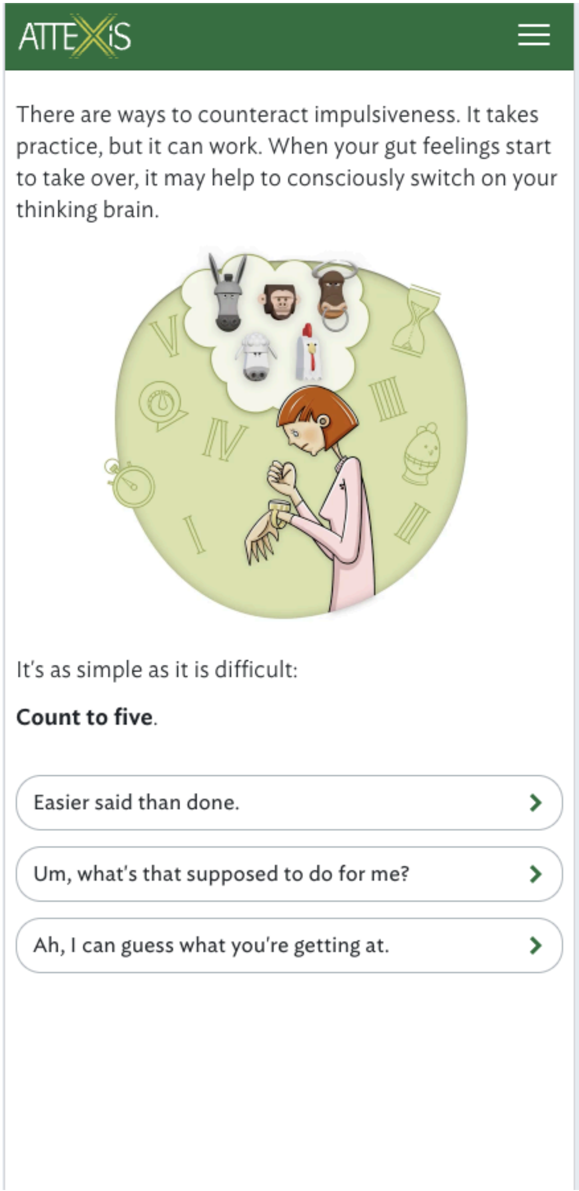

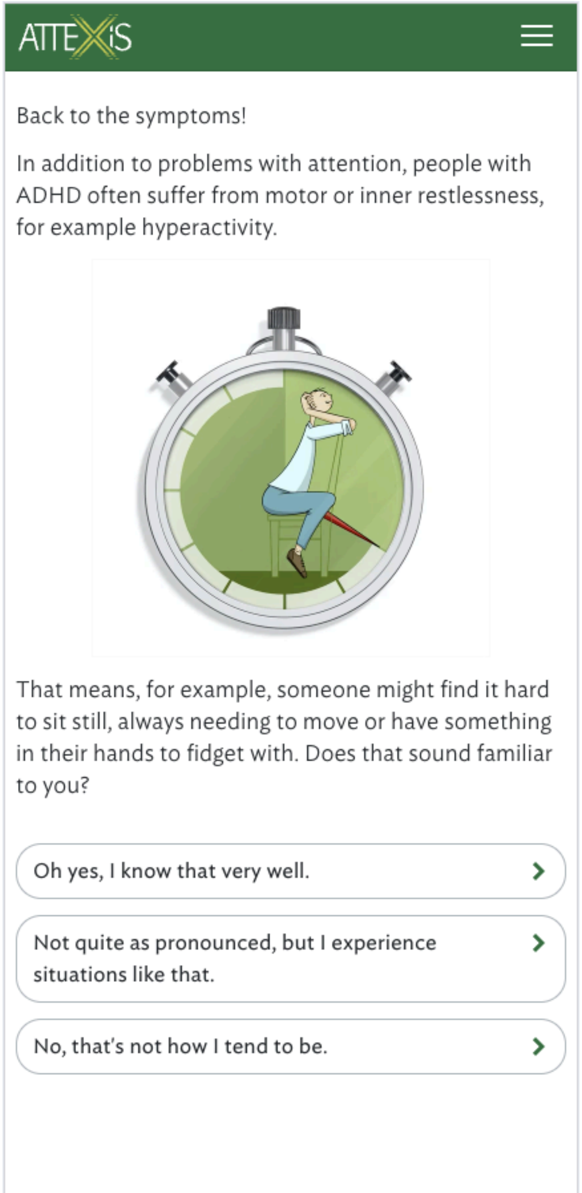

Supplementary Methods 1. Sample Size Calculation

The sample size was based on the primary outcome: ADHD symptom severity at 3 months (ASRS total score). We conservatively assumed an effect size of $d = 0.35$, informed by prior studies in adolescents (Kollins et al., 2020) and meta-analytic findings on CBT for adult ADHD (Young, Moghaddam, & Tickle, 2020). A total of 260 participants (130 per group) was required to detect this effect with 80% power and $\alpha = .05$ (two-sided). Allowing for 20% dropout, the target sample size was $n = 326$ (2×163). Calculations were performed using the *R* package *pwr* (Champely et al., 2020).

Supplementary Methods 2. Statistical Analysis

All analyses were performed with *R*, version 4.4.1 (R Core Team, 2024), following a pre-specified analysis plan. We conducted intent-to-treat (ITT) analyses under the 'missing at random' assumption as the primary analysis for all outcomes, including all randomized participants in the intervention and control group. Treatment effects at T1 were assessed using ANCOVA, adjusting for baseline values. Treatment effects are reported as baseline-adjusted mean differences with 95% CIs along with standardized effect sizes (Cohen's *d*) based on estimated marginal means (Lenth, 2024). To address missing data, we applied bootstrapped maximum likelihood multiple imputation, generating 1000 bootstrap samples, with two imputations per sample created via multivariate imputation by chained equations (MICE) using the R packages *bootImpute* and *mice* (van Buuren & Groothuis-Oudshoorn, 2011; von Hippel & Bartlett, 2021). Per-protocol (PP) analyses, defined as including only intervention participants who had registered to use *attexis*, alongside all participants in the control group, applied the same statistical procedures as the ITT analyses.

As a sensitivity analysis, we applied jump-to-reference (J2R) imputation, a conservative approach that assumes participants in the intervention group who drop out will subsequently follow the outcome trajectory of the control group from the point of dropout onward (Carpenter, Roger, & Kenward, 2013). This method reflects a 'missing not at random' assumption, where dropout is related to the treatment effect, and tends to attenuate treatment effects by modeling dropouts as if they no longer benefit from the intervention. To implement J2R, we applied bootstrapped maximum likelihood multiple imputation, generating 1000 bootstrap samples with two imputations per sample, using the R packages *bootImpute* and *mlmi* (Bartlett, 2023; von Hippel & Bartlett, 2021). ITT, J2R, and PP analyses were repeated at T2 to evaluate the durability of effects.

For assessment of the clinical relevance of results, an additional responder analysis was conducted based on ITT data for the primary endpoint, ADHD symptom severity. Responders were defined as participants who achieved a reduction of at least 30% from baseline to T1 (Buitelaar, Montgomery, & van Zwieten-Boot, 2003). For secondary endpoints, responder analyses were based on predefined minimal clinically important differences (MCIDs), where available. In the absence of an established MCID, the reliable change index (RCI) was used, calculated as the ratio of an individual's pre-post score

difference to the standard error of that difference. An RCI > 1.96 ($p < .05$) was interpreted as a clinically meaningful change (Jacobson & Truax, 1991). Responders were defined as participants who met or exceeded the respective threshold between T0 and T1. Group differences in responder rates were analyzed using χ^2 tests, and odds ratios (OR) were calculated to estimate effect sizes.

As part of the pre-specified safety analyses, symptom worsening was also assessed in patients with complete observations. Participants were classified as having experienced symptom worsening if their ASRS total score increased from baseline to T1. Group differences in the proportion of participants with symptom worsening were analyzed using a χ^2 test, along with the OR.

The following subgroup analyses were pre-specified to explore potential moderators of treatment effectiveness: (1) sex (male, female, intersex); (2) psychotherapy at baseline (yes vs. no); (3) intake of psychotropic medication at baseline (yes vs. no); and (4) changes in treatment status (i.e., psychotherapy and/or regular medication) between T0 and T1 (change vs. no change). These analyses were conducted using the ITT dataset and followed the same analytic procedures as the primary outcome analysis. An exception applied to the subgroup analysis on treatment changes between T0 and T1, where only participants with available outcome data at T1 could be included due to the nature of the comparison.

All statistical tests were considered significant at the two-sided 5% level. No correction for multiplicity was required, as a fixed-sequence gatekeeping testing strategy was employed: significance on preceding endpoints was required before subsequent endpoints could be tested (FDA, 2022). The order of secondary endpoints was pre-specified for this approach.

Supplementary Table 1. Comparison of baseline characteristics of dropouts and completers (up to the primary time point of assessment at T1). Values represent mean (SD) unless stated otherwise.

	Dropouts	Completers	Statistical comparison
	n = 21	n = 316	
**Age**	33.22 (9.01)	37.93 (9.57)	$t = -2.31, p = .030$
**Sex (n [%])**			$\chi^2 = 0.88, p = .348$
female	13 (61.9)	226 (71.5)	
male	8 (38.1)	90 (28.5)	
intersexual	0 (0.0)	0 (0.0)	
**Family situation (n [%])**			$\chi^2 = 0.80, p = .670$
never married	13 (61.9)	164 (51.9)	
married / registered civil partnership	7 (33.3)	135 (42.7)	
divorced / registered partnership annulled	1 (4.8)	17 (5.4)	
widowed / registered partner deceased	0 (0.0)	0 (0.0)	
**Education (n [%])**			$\chi^2 = 4.06, p = .541$
Hauptschulabschluss	1 (4.8)	3 (0.9)	
Realschulabschluss	2 (9.5)	19 (6.0)	
Fachhochschulreife	2 (9.5)	30 (9.5)	
Abitur (A-levels)	3 (14.3)	32 (10.1)	
completed vocational training	2 (9.5)	59 (18.7)	
completed university studies	11 (52.4)	173 (54.7)	
**Employment (n [%])**			$\chi^2 = 12.18, p = .095$
not employed	4 (19.0)	38 (12.0)	
employed irregularly	1 (4.8)	5 (1.6)	
marginal employment	1 (4.8)	11 (3.5)	
employed part-time	1 (4.8)	110 (34.8)	
employed full-time	14 (66.7)	128 (40.5)	
in vocational training	0 (0.0)	6 (1.9)	
on parental leave	0 (0.0)	12 (3.8)	
in re-training	0 (0.0)	6 (1.9)	
**Ethnicity (multiple answers possible; n [%])**			
White	16 (76.2)	297 (94.0)	$\chi^2 = 6.93, p = .008$
Black	0 (0.0)	4 (1.3)	$\chi^2 = 0, p = 1$
Middle Eastern	4 (19.0)	11 (3.5)	$\chi^2 = 7.86, p = .005$

	Dropouts	Completers	Statistical comparison
South East Asian	0 (0.0)	3 (0.9)	$\chi^2 = 0, p = 1$
Latin American	1 (4.8)	6 (1.9)	$\chi^2 = 0.01, p = .920$
Unknown	0 (0.0)	1 (0.3)	$\chi^2 = 0, p = 1$
Prefer not to say	0 (0.0)	3 (0.9)	$\chi^2 = 0, p = 1$
**Sick days (last 3 months; n [%])**			$\chi^2 = 0.82, p = .845$
0 days	11 (52.4)	149 (47.2)	
1-5 days	5 (23.8)	83 (26.3)	
6-10 days	1 (4.8)	32 (10.1)	
10+ days	4 (19.0)	52 (16.5)	
**Sick pay days (last 3 months; n [%])**			$\chi^2 = 1.95, p = .583$
0 days	21 (100.0)	289 (91.5)	
1-5 days	0 (0.0)	11 (3.5)	
6-10 days	0 (0.0)	3 (0.9)	
10+ days	0 (0.0)	13 (4.1)	
**Prior ADHD diagnosis (n [%])**	13 (61.9)	137 (43.4)	$\chi^2 = 2.74, p = .098$
**Age at diagnosis (in individuals with prior ADHD diagnosis; in years)**	28.38 (13.85)	33.65 (12.56)	$t = -1.32, p = .208$
**Current psychiatric diagnoses (Mini-DIPS) (multiple answers possible; n [%])**			
**Anxiety disorders**			
Panic disorder	0 (0.0)	7 (2.2)	$\chi^2 = 0, p = 1$
Agoraphobia	0 (0.0)	11 (3.5)	$\chi^2 = 0.06, p = .814$
Specific phobia	5 (23.8)	33 (10.4)	$\chi^2 = 2.31, p = .129$
Social anxiety disorder	3 (14.3)	36 (11.4)	$\chi^2 = 0.00, p = .961$
Generalized anxiety disorder	0 (0.0)	14 (4.4)	$\chi^2 = 0.18, p = 0.674$
**Mood disorders**			
Bipolar disorder	0 (0.0)	1 (0.3)	$\chi^2 = 0, p = 1$
Major depressive disorder	4 (19.0)	86 (27.2)	$\chi^2 = 0.32, p = .572$
Persistent depressive disorder	0 (0.0)	4 (1.3)	$\chi^2 = 0, p = 1$
**Sleep-wake disorders**			
Hypersomnia	2 (9.5)	6 (1.9)	$\chi^2 = 2.20, p = .138$
Insomnia	0 (0.0)	24 (7.6)	$\chi^2 = 0.76, p = .383$
**Currently in psychotherapy (n [%])**	7 (33.3)	72 (22.8)	$\chi^2 = 1.22, p = .269$

	Dropouts	Completers	Statistical comparison
**Number of psychotherapy sessions[a]**	1.33 (2.27)	1.44 (3.16)	t = -0.20, p = .841
**Currently treated by ADHD specialist**	3 (14.3)	9 (2.8)	χ^2 = 4.54, p = .033
**Ever in psychotherapy (n [%])**	10 (47.6)	188 (59.5)	χ^2 = 1.15, p = .284
**Self-medicating (n[%])**	1 (4.8)	25 (7.9)	χ^2 = 0.01, p = .919
**Currently taking any psychotropic medication[b] (n [%])**	12 (57.1)	110 (34.8)	χ^2 = 4.25, p = .039
**Regular medication (multiple answers possible; n [%])**			
Antipsychotics	0 (0.0)	1 (0.3)	χ^2 = 0, p = 1
Anxiolytics	0 (0.0)	0 (0.0)	
Hypnotics and sedatives	0 (0.0)	3 (0.9)	χ^2 = 0, p = 1
Antidepressants	4 (19.0)	31 (9.8)	χ^2 = 0.95, p = .330
Psychostimulants	8 (38.1)	85 (26.9)	χ^2 = 1.24, p = .266
**Medication as needed (multiple answers possible; n [%])**			
Antipsychotics	0 (0.0)	0 (0.0)	
Anxiolytics	0 (0.0)	0 (0.0)	
Hypnotics and sedatives	0 (0.0)	4 (1.3)	χ^2 = 0, p = 1
Antidepressants	0 (0.0)	3 (0.9)	χ^2 = 0, p = 1
Psychostimulants	4 (19.0)	15 (4.7)	χ^2 = 5.12, p = .024
**ASRS total score**	57.2 (6.9)	52.2 (7.2)	t = 3.27, p = .003
**WSAS total score**	27.4 (7.9)	22.8 (7.1)	t = 2.65, p = .015
**PHQ-9 total score**	14.0 (5.8)	11.6 (4.6)	t = 1.81, p = .085
**RSES total score**	16.7 (5.7)	17.2 (5.8)	t = -0.42, p = .675
**AQoL-8D total score**	60.8 (12.8)	63.4 (9.5)	t = -0.92, p = .370

[a] Calculated across all participants, including those with zero sessions.
[b] Including medications classified under the Anatomical Therapeutic Chemical (ATC) system as N05 (psycholeptics) and N06 (psychoanaleptics).
Abbreviations: AQoL-8D = Assessment of Quality of Life - 8 Dimensions; ASRS = Adult ADHD Self-Report Scale; PHQ-9: Patient Health Questionnaire-9; RSES = Rosenberg Self-Esteem Scale; WSAS = Work and Social Adjustment Scale.

Supplementary Table 2. Results of primary and secondary endpoints for per protocol (PP) analyses.

	**Time**	**Control**			***attexis***			**ANCOVA**		
		n	mean	SD	n	mean	SD	Treatment effect[a] (95% CI)	*p*-value	Cohen's *d* (95% CI)[b]
ASRS	T0	173	52.7	7.1	163	52.3	7.4	-	-	-
	T1	173	49.9	7.6	163	44.6	8.4	-5.0 (-6.4, -3.6)	< .001	0.86 (0.63, 1.08)
	T2	173	48.5	8.5	163	43.6	8.7	-4.6 (-6.2, -2.9)	< .001	0.62 (0.39, 0.84)
WSAS	T0	173	22.8	7.1	163	23.2	7.2	-	-	-
	T1	173	22.9	7.0	163	19.5	7.2	-3.7 (-5.0, -2.4)	< .001	0.62 (0.39, 0.85)
	T2	173	21.3	7.4	163	18.3	7.7	-3.2 (-4.7, -1.7)	< .001	0.48 (0.26, 0.70)
PHQ-9	T0	173	11.8	4.4	163	11.7	5.0	-	-	-
	T1	173	10.4	4.1	163	9.3	4.1	-1.1 (-1.8, -0.3)	.004	0.31 (0.09, 0.53)
	T2	173	10.6	4.6	163	9.1	4.2	-1.5 (-2.4, -0.6)	.001	0.36 (0.14, 0.59)
RSES	T0	173	17.2	5.7	163	17.0	5.9	-	-	-
	T1	173	17.6	5.6	163	19.2	5.6	1.7 (0.9, 2.6)	< .001	0.48 (0.24, 0.71)
	T2	173	18.1	6.0	163	19.9	5.9	1.9 (0.9, 2.9)	< .001	0.43 (0.2, 0.66)
AQoL-8D	T0	173	63.3	9.5	163	63.0	10.0	-	-	-
	T1	173	64.2	9.1	163	66.7	9.9	2.6 (1.3, 4.0)	< .001	0.44 (0.21, 0.68)
	T2	173	64.7	10.3	163	68.3	10.2	3.8 (2.1, 5.4)	< .001	0.49 (0.27, 0.70)

[a] Between-group difference on the original scale at 3 months (T1) and 6 months (T2), adjusted for baseline scores.

[b] Based on baseline-adjusted means; positive values show effects in favor of the intervention group.

Abbreviations: AQoL-8D = Assessment of Quality of Life - 8 Dimensions; ASRS = Adult ADHD Self-Report Scale; PHQ-9: Patient Health Questionnaire-9; RSES = Rosenberg Self-Esteem Scale; WSAS = Work and Social Adjustment Scale.

Supplementary Table 3. Results of primary and secondary endpoints for jump-to-reference (J2R) sensitivity analyses.

	Time	Control			*attexis*			ANCOVA		
		n	mean	SD	n	mean	SD	Treatment effect[a] (95% CI)	*p*-value	Cohen's *d* (95% CI)[b]
ASRS	T0	173	52.7	7.1	164	52.2	7.4	-	-	-
	T1	173	49.9	7.5	164	45.1	8.7	-4.4 (-5.7, -3.2)	< .001	0.75 (0.55, 0.95)
	T2	173	48.5	8.4	164	44.6	8.9	-3.6 (-5.1, -2.2)	< .001	0.49 (0.30, 0.69)
WSAS	T0	173	22.8	7.1	164	23.3	7.3	-	-	-
	T1	173	22.9	7.1	164	19.9	7.5	-3.3 (-4.6, -2.1)	< .001	0.55 (0.34, 0.76)
	T2	173	21.3	7.2	164	18.7	7.9	-2.8 (-4.1, -1.5)	< .001	0.43 (0.23, 0.62)
PHQ-9	T0	173	11.8	4.4	164	11.7	5.0	-	-	-
	T1	173	10.4	4.1	164	9.3	4.1	-1.0 (-1.7, -0.3)	.005	0.29 (0.08, 0.49)
	T2	173	10.6	4.5	164	9.2	4.3	-1.3 (-2.1, -0.5)	.001	0.32 (0.13, 0.51)
RSES	T0	173	17.2	5.7	164	17.1	5.9	-	-	-
	T1	173	17.6	5.6	164	19.1	5.6	1.5 (0.8, 2.3)	< .001	0.42 (0.21, 0.63)
	T2	173	18.1	5.9	164	19.8	6.0	1.8 (0.9, 2.7)	< .001	0.42 (0.22, 0.61)
AQoL-8D	T0	173	63.3	9.5	164	63.1	10.0	-	-	-
	T1	173	64.3	9.0	164	66.5	9.9	2.4 (1.2, 3.6)	< .001	0.40 (0.2, 0.6)
	T2	173	64.6	10.2	164	67.8	10.4	3.3 (1.8, 4.8)	< .001	0.44 (0.25, 0.63)

[a]Between-group difference on the original scale at 3 months (T1) and 6 months (T2), adjusted for baseline scores.

[b] Based on baseline-adjusted means; positive values show effects in favor of the intervention group.

Abbreviations: AQoL-8D = Assessment of Quality of Life - 8 Dimensions; ASRS = Adult ADHD Self-Report Scale; PHQ-9: Patient Health Questionnaire-9; RSES = Rosenberg Self-Esteem Scale; WSAS = Work and Social Adjustment Scale.

Supplementary Table 4. Subgroup analysis based on sex for the primary endpoint ADHD symptom severity (assessed with the ASRS total score) at T1.

Time		Control			*attexis*			ANCOVA		
		n	mean	SD	n	mean	SD	Treatment effect (95% CI)[a]	*p*-value	Cohen's *d* (95% CI)[b]
Women (n = 239)	T0	118	52.9	6.7	121	52.7	7.0	-	-	-
	T1	118	50.1	7.1	121	45.4	8.3	-4.5 (-6.1, -3.0)	< .001	0.81 (0.55, 1.07)
Men (n = 98)	T0	55	52.2	7.7	43	51.0	8.3	-	-	-
	T1	55	49.6	8.5	43	42.4	8.2	-6.3 (-9.0, -3.6)	< .001	1.01 (0.59, 1.44)

[a] Between-group difference on the original scale at 3 months (T1), adjusted for baseline scores.
[b] Based on baseline-adjusted means; positive values show effects in favor of the intervention group.

Supplementary Table 5. Subgroup analysis based on psychotherapy status at baseline for the primary endpoint ADHD symptom severity (assessed with the ASRS total score) at T1.

Time		Control			*attexis*			ANCOVA		
		n	mean	SD	n	mean	SD	Treatment effect (95% CI)[a]	*p*-value	Cohen's *d* (95% CI)[b]
In psycho-therapy (n = 79)	T0	43	52.6	7.0	36	50.4	7.0	-	-	-
	T1	43	49.0	7.1	36	42.0	8.3	-5.3 (-8.0, -2.6)	< .001	0.98 (0.48, 1.47)
Not in psycho-therapy (n = 258)	T0	130	52.8	7.1	128	52.8	7.4	-	-	-
	T1	130	50.3	7.7	128	45.4	8.2	-4.9 (-6.5, -3.3)	< .001	0.83 (0.58, 1.09)

[a] Between-group difference on the original scale at 3 months (T1), adjusted for baseline scores.
[b] based on baseline-adjusted means; positive values show effects in favor of the intervention group.

Supplementary Table 6. Subgroup analysis based on psychotropic medication at baseline for the primary endpoint ADHD symptom severity (assessed with the ASRS total score) at T1.

	**Time**	**Control**			***attexis***			**ANCOVA**		
		n	mean	SD	n	mean	SD	Treatment effect (95% CI)[a]	*p*-value	Cohen's *d* (95% CI)[b]
On medication[c] (n = 122)	T0	59	52.8	6.8	63	51.6	7.5	-	-	-
	T1	59	49.6	7.5	63	44.6	8.5	-4.1 (-6.4, -1.8)	< .001	0.69 (0.30, 1.08)
Not on medication[c] (n = 215)	T0	114	52.7	7.2	101	52.6	7.3	-	-	-
	T1	114	50.1	7.6	101	44.6	8.3	-5.5 (-7.1, -3.8)	< .001	0.96 (0.68, 1.23)

[a] Between-group difference on the original scale at 3 months (T1), adjusted for baseline scores.

[b] based on baseline-adjusted means; positive values show effects in favor of the intervention group.

[c] Including medications classified under the Anatomical Therapeutic Chemical (ATC) system as N05 (psycholeptics) and N06 (psychoanaleptics).

Supplementary Table 7. Subgroup analysis based on changes in treatment from baseline to T1 for the primary endpoint ADHD symptom severity (assessed with the ASRS total score) at T1[a].

	Time	Control			*attexis*			ANCOVA		
		n	mean	SD	n	mean	SD	Treatment effect (95% CI)[b]	*p*-value	Cohen's *d* (95% CI)[c]
Changes in treat-ment (n = 53)	T0	31	53.5	6.6	22	51.2	5.8	-	-	-
	T1	31	50.3	7.5	22	43.7	7.1	-4.5 (-7.3, -1.7)	< .001	0.93 (0.33, 1.52)
No changes in treat-ment (n = 263)	T0	136	52.3	7.1	127	51.8	7.6	-	-	-
	T1	136	49.7	7.6	127	44.4	8.6	-4.9 (-6.4, -3.5)	< .001	0.82 (0.56, 1.07)

[a] Due to the nature of the analysis, only participants with complete observations were included in the subgroup analysis of changes in treatment from T0 to T1.
[b] Between-group difference on the original scale at 3 months (T1), adjusted for baseline scores.
[c] Based on baseline-adjusted means; positive values show effects in favor of the intervention group.

Supplementary References

Bartlett, J. W. (2023). Reference-Based Multiple Imputation–What is the Right Variance and How to Estimate It. *Statistics in Biopharmaceutical Research*, *15*(1), 178–186. https://doi.org/10.1080/19466315.2021.1983455

Buitelaar, J. K., Montgomery, S. A., & van Zwieten-Boot, B. J. (2003). Attention deficit hyperactivity disorder: Guidelines for investigating efficacy of pharmacological intervention. *European Neuropsychopharmacology*, *13*(4), 297–304. https://doi.org/10.1016/S0924-977X(03)00047-6

Carpenter, J. R., Roger, J. H., & Kenward, M. G. (2013). Analysis of Longitudinal Trials with Protocol Deviation: A Framework for Relevant, Accessible Assumptions, and Inference via Multiple Imputation. *Journal of Biopharmaceutical Statistics*, *23*(6), 1352–1371. https://doi.org/10.1080/10543406.2013.834911

Champely, S., Ekstrom, C., Dalgaard, P., Gill, J., Weibelzahl, S., Anandkumar, A., … De Rosario, H. (2020). *pwr: Basic functions for power analysis*. Retrieved from https://cran.r-project.org/web/packages/pwr/

FDA. (2022). *Multiple Endpoints in Clinical Trials Guidance for Industry*. Center for Drug Evaluation and Research Center for Biologics Evaluation and Research. Retrieved from https://www.fda.gov/regulatory-information/search-fda-guidance-documents/multiple-endpoints-clinical-trials-guidance-industry

Jacobson, N. S., & Truax, P. (1991). Clinical significance: A statistical approach to defining meaningful change in psychotherapy research. *Journal of Consulting and Clinical Psychology*, *59*(1), 12–19. https://doi.org/10.1037//0022-006x.59.1.12

Kollins, S. H., DeLoss, D. J., Cañadas, E., Lutz, J., Findling, R. L., Keefe, R. S. E., … Faraone, S. V. (2020). A novel digital intervention for actively reducing severity of

paediatric ADHD (STARS-ADHD): A randomised controlled trial. *The Lancet Digital Health*, *2*(4), e168–e178. https://doi.org/10.1016/S2589-7500(20)30017-0

Lenth, R. (2024). *Emmeans: Estimated Marginal Means, aka Least-Squares Means. R package version 1.10.5*. Retrieved from https://rvlenth.github.io/emmeans/.

R Core Team. (2024). *R: A language and environment for statistical computing*. Vienna, Austria. Retrieved from https://cran.r-project.org/

van Buuren, S., & Groothuis-Oudshoorn, K. (2011). mice: Multivariate Imputation by Chained Equations in R. *Journal of Statistical Software*, *045*(i03). https://doi.org/10.18637/jss.v045.i03

von Hippel, P. T., & Bartlett, J. W. (2021). Maximum likelihood multiple imputation: Faster imputations and consistent standard errors without posterior draws. *Statistical Science*, *36*(3), 400–420. https://doi.org/10.1214/20-STS793

Young, Z., Moghaddam, N., & Tickle, A. (2020). The Efficacy of Cognitive Behavioral Therapy for Adults With ADHD: A Systematic Review and Meta-Analysis of Randomized Controlled Trials. *Journal of Attention Disorders*, *24*(6), 875–888. https://doi.org/10.1177/1087054716664413
